# Antiemetic efficacy and safety of granisetron or palonosetron alone and in combination with a corticosteroid for ABVD therapy-induced nausea and vomiting

**DOI:** 10.1186/s40780-017-0097-4

**Published:** 2018-01-09

**Authors:** Mayako Uchida, Tsutomu Nakamura, Kojiro Hata, Hiroyuki Watanabe, Yasuo Mori, Koji Kato, Kenjiro Kamezaki, Katsuto Takenaka, Motoaki Shiratsuchi, Keiko Hosohata, Toshihiro Miyamoto, Koichi Akashi

**Affiliations:** 1 Department of Pharmacy, Kyushu University Hospital, 3-1-1, Maidashi, Higashi-ku, Fukuoka, 812-8582 Japan; 2 Education and Research Center for Clinical Pharmacy, Osaka University of Pharmaceutical Sciences, 4-20-1 Nasahara, Takatsuki, Osaka, 569-1094 Japan; 3 Department of Medicine and Biosystemic Science, Kyushu University Graduate School of Medical Sciences, 3-1-1, Maidashi, Higashi-ku, Fukuoka City, 812-8582 Japan; 4 Department of Medicine and Bioregulatory Science, Kyushu University Graduate School of Medical Sciences, 3-1-1, Maidashi, Higashi-ku, Fukuoka City, 812-8582 Japan

**Keywords:** Granisetron, Palonosetron, Chemotherapy-induced nausea and vomiting, Complete response, Delayed phase, Corticosteroid

## Abstract

**Background:**

Antiemetic effects and safety of granisetron or palonosetron alone and in combination with a corticosteroid against chemotherapy-induced nausea and vomiting (CINV) were retrospectively evaluated in patients with Hodgkin lymphoma receiving adriamycin, bleomycin, vinblastine, and dacarbazine (ABVD) therapy.

**Methods:**

A total of 39 patients were eligible for this study. Before ABVD therapy, granisetron or palonosetron was intravenously administered with or without a corticosteroid (dexamethasone or hydrocortisone) and aprepitant. The proportions of patients with complete control (CC) during the overall (0–120 h after the start of ABVD therapy), acute (0–24 h) and delayed (24–120 h) phases were evaluated. CC was defined as no vomiting and no use of antiemetic rescue medication with only grade 0–1 nausea.

**Results:**

Granisetron and palonosetron were administered in 21 and 18 patients, respectively. The CC rate during the acute, delayed and overall phases was not statistically different between the two groups. The CINV was completely controlled during overall phase in 58.3% of patients receiving granisetron or palonosetron in combination with a corticosteroid, whereas in 11.1% of those without co-treatment of a corticosteroid (*P* < 0.05). There were significantly higher frequencies of anorexia, leucopenia and neutropenia in the palonosetron group. There is a statistically significant difference in the frequency of febrile neutropenia between presence and absence of a corticosteroid (*p* = 0.024).

**Conclusion:**

These findings suggested that a combination use of a corticosteroid with a 5-HT_3_ receptor antagonist was preferable for CINV control in patients with Hodgkin lymphoma receiving ABVD therapy, although the careful management of febrile neutropenia is required.

**Trial registration:**

The study approval numbers in the institution; 24–12 and 24–359. Registered April 17, 2012 and June 21, 2012.

## Background

Hodgkin lymphoma is a type of lymphoma, which is a blood cancer that starts in the lymphatic system. Adriamycin, bleomycin, vinblastine, and dacarbazine (ABVD) therapy is a representative standard therapy for Hodgkin lymphoma. Chemotherapy can commonly induces nausea and vomiting, which are severe problematic adverse drug events (ADEs) for patients with cancer [1]. Therefore, to control chemotherapy-induced nausea and vomiting (CINV) adequately is very important for improving quality of life of cancer patients.

According to some antiemetic guidelines, ABVD therapy is classified as highly emetogenic chemotherapy (HEC) [2–4]. The use of a serotonin (5-hydroxytryptamine; 5-HT) type 3 (5-HT_3_) receptor antagonist, such as granisetron and palonosetron, in combination with dexamethasone and aprepitant is recommended for antiemetic management of CINV with HECs. CINV is often classified as either acute or delayed, which are defined as occurring within 24 h and more than 24 h after the start of the chemotherapy, respectively. The first-generation 5-HT_3_ receptor antagonist, such as granisetron, showed a highly prophylactic effect against acute CINV, but demonstrated less effect against delayed CINV [5]. On the contrary, it has been reported that the second-generation palonosetron had the antiemetic effects superior to those of the first-generation 5-HT_3_ receptor antagonists ondansetron [6, 7] and dolasetron [8] during the overall, acute, and delayed phases. In our present study, the ability of palonosetron compared to granisetron to appropriately suppressed delayed CINV in patients receiving rituximab, cyclophosphamide, doxorubicin, vincristine, and prednisolone in combination therapy therapy [9], although it is unclear which is more effective against CINV associated with ABVD therapy, granisetron- or palonosetron-based antiemetic treatment.

Meanwhile, in patients with Hodgkin lymphoma, the lymphocytes grow abnormally and spread beyond the lymphatic system, although which helps the immune system get rid of waste and fight infections under normal conditions. It is considered that most patients receiving ABVD therapy are in a state of immunosuppression [10, 11], and so the administration of corticosteroids including dexamethasone for the prevention of CINV may lead to excess immunosuppression. However, it remains unclear whether no use of corticosteroids influence the antiemetic efficacy and the frequency of ADEs in patients with Hodgkin lymphoma receiving ABVD therapy.

In the present study, the effect and safety of the antiemetic regimens with or without a corticosteroids against CINV in patients with Hodgkin lymphoma receiving ABVD therapy were assessed, and those were also compared between granisetron and palonosetron which are first- and second-generation 5-HT_3_ receptor antagonists, respectively.

## Methods

### Patients

In the present study, eligible participants were patients aged between 20 and 65 years who received ABVD chemotherapy as the treatment for Hodgkin lymphomas in the Department of Hematology, Kyushu University Hospital (April 2007 to December 2015). Patients were excluded if they had hyponatremia, hypercalcemia, adrenal metastasis, or an Eastern Cooperative Oncology Group performance status (ECOG-PS) score of more than 2; had nausea and/or vomiting in the last 24 h prior to treatment initiation; or were taking laxative agents and antiemetic drugs including olanzapine and lorazepam.

### Dosage and administration

The ABVD therapy regimen is shown in Table 1. Adriamycin (25 mg/m^2^) was administered on days 1 and 15. Bleomycin (10 mg/m^2^ up to 15 mg/body), vinblastine (6 mg/m^2^ up to 10 mg/body), and dacarbazine (375 mg/m^2^) were administered on days 1 and 15. Twenty-eight days were defined as one course. Granisetron (3 mg/body) or palonosetron (0.75 mg/body) were intravenously administered 30 min before the initiation of ABVD therapy. Dexamethasone was administered at a dose of 3.3, 6.6 or 8.25 mg in 3 patients on days 1 and 15, and hydrocortisone (100 mg) was administered on days 1 and 15 in 9 patients. The administration of the corticosteroids were carried out intravenously 30 min prior to chemotherapy treatment once daily on days 1 and 15. Aprepitant was orally administered at a dose of 125 mg once daily one hour before start of chemotherapy on days 1 and at a dose of 80 mg once daily on days 2 and 3 in the morning for each cycle. Metoclopramide, prochlorperazine, haloperidol, and/or hydroxyzine were used as rescue medications.

**Table 1:** ABVD therapy regimen

Drugs^a,b^	Daily dosage	Route of administration	Timing of administration
Adriamycin	25 mg/m^2^	15-min i.v. infusion	once daily on days 1 and 15
Bleomycin^c^	10 mg/m^2^	15-min i.v. infusion	once daily on days 1 and 15
Vinblastine^d^	6 mg/m^2^	15-min i.v. infusion	once daily on days 1 and 15
Dacarbazine	375 mg/m^2^	2-h i.v. infusion	once daily on days 1 and 15

*i.v*. intravenously

^a^Granisetron (3 mg/body) was intravenously administered on days 1 and 15

^b^Palonosetron (0.75 mg/body) was intravenously administered on days 1 and 15

^c^Maximum daily dose was 15 mg

^d^Maximum daily dose was 10 mg

### Data collection and assessment

All data were collected from the electronic medical record system. The data after the second course were excluded in order to remove confounding factors such as predictive nausea and vomiting. The occurrence of nausea, vomiting, or use of rescue medication during the overall (0 to 120 h after the start of ABVD therapy), acute (0 to 24 h), and delayed (24 to 120 h) phases were assessed. ADEs including nausea and vomiting, were monitored during the overall study period and described in the electronic medical record system twice a day (morning and evening) by doctors, nurses, and pharmacists, according to the Common Terminology Criteria for Adverse Events (CTCAE) v.4.0.

This study was conducted in accordance with the Declaration of Helsinki and its amendments.

The endpoint of this study was the proportion of patients with complete control (CC) during the overall, acute, and delayed phases. CC was defined as complete response (CR) and no more than mild nausea (grade 0 or 1).

### Statistical analysis

Statistical analysis was performed using JMP13 software (SAS Institute Inc. Cary, NC, USA). Fisher’s exact test was used to examine differences in frequencies of categorical data between the granisetron and palonosetron groups. The statistical significance of the difference between the median values of age was calculated with the Mann-Whitney U-test. In the univariate analysis, sex, age, ECOG-PS, and use of granisetron, palonosetron, a corticosteroid and aprepitant were chosen as variables. The factors with *p* values < 0.10 in univariate analyses were included in a stepwise multivariate logistic regression analysis with backward selection. Two-tailed p values of less than 0.05 were considered to indicate a statistically significant difference.

## Results

### Patient baseline clinical characteristics

A total of 44 patients participated, but 5 patients were excluded from the study because they failed to meet inclusion criteria or had missing data. The patients’ baseline clinical characteristics are shown in Table 2. There was no significant difference in the variables between the granisetron and palonosetron groups.

**Table 2:** Patient characteristics

Variable	Granisetron (*n* = 21)	Palonosetron (*n* = 18)	*p* value
Number of patients	21	18	
Sex			
Male	11	5	0.192
Female	10	13	
Age			
Median, year (range)	30 (20–55)	31 (25–58)	0.863
Age (years)			
≥ 35	8	8	0.752
< 35	13	10	
ECOG-PS^a^ score			
0	20	16	0.586
1	1	2	
Combined antiemetics			
None	6	5	0.633
Corticosteroid^b^	0	2	
Aprepitant	9	7	
Corticosteroid + Aprepitant	6	4	

^a^ECOG-PS, Eastern Cooperative Oncology Group performance status

^b^Corticosteroids included dexamethasone or hydrocortisone

### Antiemetic effects

**Fig. 1:**
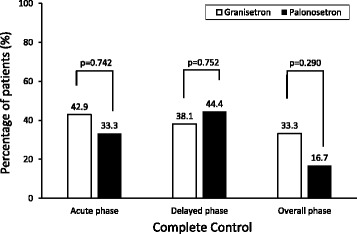
Complete control by phase. The bar shows the percentage of patients achieving complete control (CC) during the acute (0–24 h after initiation of chemotherapy), delayed (24–120 h) and overall phases. CC was defined as no vomiting and no use of antiemetic rescue medication with only grade 0–1 nausea. White and black bars represent the granisetron- and palonosetron-based antiemetic regimens, respectively

**Fig. 2:**
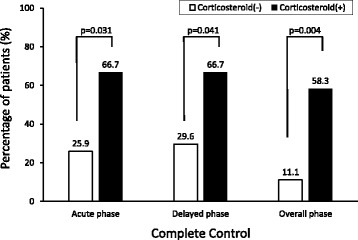
Effect of co-treatment with a corticosteroid on the rate of complete control by phase. The bar shows the percentage of patients achieving complete control (CC) during the acute (0–24 h after initiation of chemotherapy), delayed (24–120 h) and overall phases. CC was defined as no vomiting and no use of antiemetic rescue medication with only grade 0–1 nausea. White and black bars represent the absence and presence of a corticosteroid, respectively. Corticosteroids included dexamethasone or hydrocortisone

In 38.5% of 39 patients receiving ABVD therapy, CINV was completely controlled by treatment with granisetron or palonosetron during the overall study period, although the nausea and vomiting in 24 patients were not. The CC rates were not significantly different between the granisetron and palonosetron groups during the overall, acute, and delayed phases (Fig. 1). The univariate analyses were performed to detect factors influencing CINV control. When a corticosteroid was combined as antiemetic agents, the CC rates were significantly higher than those in the group without a corticosteroid throughout the study period (Fig. 2). Thirty-five years of age and above was also a significant factor that improved the CC rate during the delayed phase, and multivariate logistic regression analysis showed that corticosteroid use (odds ratio, 5.4; 95% confidence interval, 1.0 to 29.1) and 35 years of age and above (odds ratio, 8.7; 95% confidence interval, 1.8 to 42.2) were independent factors associated with complete control of CINV. Sex, ECOG-PS, generation of 5-HT_3_ receptor antagonist, and aprepitant use were not identified as a significant influencing factor during any of the phases.

### Adverse drug events (ADEs)

The numbers of patients experiencing side effects (SEs) in the granisetron and palonosetron groups are shown in Table 3. The most frequent SEs was anorexia and it occurred in more than 70% patients in both the granisetron and palonosetron groups. Over half of the patients in the palonosetron group experienced anorexia, malaise, leucopenia, and neutropenia, and the frequencies of anorexia, leucopenia and neutropenia were significantly higher than those of the SEs in the granisetron group. There was also statistical significant difference in the frequency of malaise between the two groups. Fever and oral mucositis and were also observed at the frequencies of more than 10% in both the granisetron and palonosetron groups, although there was not statistically significant difference. Febrile neutropenia occurred when the patients were treated with the antiemetics in combination with granisetron (Table 3). All these patients was also treated with a corticosteroid. There was a statistically significant difference in the frequency of febrile neutropenia between the antiemetic treatments with or without a corticosteroid (*p* = 0.024). No statistically significant difference was detected in the frequencies of SEs other than febrile neutropenia between the two groups. The addition of aprepitant to granisetron- or palonosetron-based antiemetic therapy did not significantly produce any increase in the frequency of SEs.

**Table 3:** Number of patients experiencing SEs in palonosetron and granisetron groups

SEs^a^	Granisetron (*n* = 21)	Palonosetron (*n* = 18)	*p* value^b^
Anorexia	15 (71.4%)	18 (100%)	0.022
Malaise	8 (38.1%)	13 (72.2%)	0.054
Leucopenia	4 (19.1%)	12 (66.7%)	0.004
Neutropenia	3 (14.3%)	12 (66.7%)	0.001
Fever	3 (14.3%)	6 (33.3%)	0.255
Oral mucositis	3 (14.3%)	3 (16.7%)	1.000
Febrile Neutropenia	3 (14.3%)	0 (0%)	0.235
Constipation	2 (9.5%)	5 (27.8%)	0.216
Headache	2 (9.5%)	2 (11.1%)	1.000
Neuropathy	2 (9.5%)	0 (0%)	0.490
Diarrhea	2 (9.5%)	0 (0%)	0.490

^a^Side effects (SEs) with frequencies of more than 5% in either group are listed

^b^A significant difference is in italics

## Discussion

The ABVD regimen includes dacarbazine, which is a classified as a HEC agent [2–4]. The ABVD therapy caused severe nausea and vomiting in about 50% of patients before the use of antiemetic agents is recommended [12], and thereby patients may refuse to complete its planned cycles [13]. To date, it has been reported that antiemetic regimens including the first-generation 5-HT_3_ receptor antagonists, ondansetron and granisetron, were more effective for CINV control in patients with Hodgkin and non-Hodgkin lymphomas receiving moderately emetogenic chemotherapy (MEC), compared to metoclopramide-based antiemetic regimens [14, 15]. In patients with HEC, to prevent CINV induced by ABVD therapy, triple antiemetic therapy (a 5-HT_3_ receptor antagonist, dexamethasone, aprepitant) is recommended, although the data regarding the antiemetic effects of these medications are not entirely sufficient for patients with hematological malignancies. In the present study, dexamethasone was used as a corticosteroid in three patients. One patient treated with dexamethasone in combination with palonosetron and aprepitant achieved CC, whereas the other two treated with dexamethasone and granisetron did not. The superiority of palonosetron to the first generation 5-HT_3_ receptor antagonists has been reported from the viewpoint of antiemetic prophylaxis for acute, delayed and overall phases in populations of patients of whom most had solid cancer [6, 7, 16–18]. However, it remains unclear which of first- or second-generation 5-HT_3_ receptor antagonists are more effective in a triple antiemetic therapy in Hodgkin lymphoma patients receiving ABVD therapy, and future large scale studies are required to address this issue.

Dexamethasone is recommended for the prevention of delayed emesis following HEC and MEC [19]. Though it seems that clinical interest has trended toward the reduction of steroid use [20–23]. In the present study, the antiemetic treatment of dexamethasone or hydrocortisone in combination of a 5-HT_3_ receptor antagonist improved the CC rate during acute, delayed and overall phases, compared to the therapy without corticosteroids (Fig. 2). This suggested that an addition of a corticosteroid was more effective in preventing CINV in the patients with Hodgkin Lymphoma receiving ABVD therapy. Meanwhile, of concern is the possibility that the risk of infection is elevated by use of steroids. During the observation period, there was a statistically significant difference in frequencies of febrile neutropenia between presence and absence of a combination of a corticosteroid (25% and 0%, respectively, *p* = 0.024). All patients with febrile neutropenia were treated with granisetron as a 5-HT_3_ receptor antagonist, but its frequency was not statistically significant different from that in those treated with palonosetron (Table 3). Meanwhile, fever and oral mucositis were also observed in both the granisetron and palonosetron groups (Table 3). The ADEs observed during the study period were possibly attributed to the ABVD therapy as well as the 5-HT_3_ receptor antagonist-based antiemetic therapy, although the extent of which remains unclear. Further investigations with relatively large number of subjects should be undertaken to confirm these preliminary results. Collectively, palonosetron or granisetron in combination with a corticosteroid could control CINV effectively during the cycles of ABVD treatment, although the careful management of febrile neutropenia is required.

Aprepitant has the potential to inhibit cytochrome P450 (CYP) 3A4 [24]. It may influence the pharmacokinetics of the drugs including vinblastine and corticosteroids, which are metabolized by CYP 3A4, and thereby increase the risk of ADEs during ABVD therapy and CINV management [25, 26]. In the present study, univariate analysis found no significant association of aprepitant use, unlike to corticosteroid use, with the CC rate and the frequencies of the ADEs during the study period. Therefore, we considered that the drug-interaction did not considerably influence CINV control during ABVD therapy.

To date, it has been reported that younger age, female sex, and/or no alcohol intake were risk factors reducing the degree of response to antiemetic therapy including ondansetron (a 5-HT_3_ receptor antagonist), dexamethasone, and aprepitant during cancer chemotherapy [27, 28]. In the present study, the multivariate logistic regression analysis showed that age less than 35 years, but not female sex, was an independent significant factor complicating CINV management during the delayed phase. The cause of the discrepancy between the results was unclear, although it might be attributed to the difference in chemotherapy and antiemetic regimens. Our present pilot study was limited by small sample size and lack of racial and ethnic diversity, and further studies are required to address these issues.

Regarding treatment-related SEs, the frequency of anorexia, leucopenia, neutropenia and stomatitis in the palonosetron group was significantly higher than that in the granisetron group in the present study (Table 3). This was almost consistent with the result of our previous report in patients with malignant lymphoma receiving first-line rituximab, cyclophosphamide, doxorubicin, vincristine, and prednisone [9]. The interaction of a 5-HT_3_ receptor antagonist with its target receptor families can affect the peripheral and central nervous system and the immune responses as well [29–31], possibly associated with the above-mentioned SEs. Besides, palonosetron has a higher binding affinity for the 5-HT_3_ receptor and a longer half-life, compared to those of granisetron [32]. Therefore the difference in the frequencies of the SEs between the granisetron and palonosetron groups may reflect the difference in the pharmacological and pharmacokinetic properties of these two antiemetics, although further studies are warranted. As mentioned above, there is no statistically difference in the antiemetic efficacy between granisetron- and palonosetron-containing regimens. Considering these findings together, granisetron may be more adequate for CINV control in patients with Hodgkin lymphoma receiving ABVD therapy, although it was needed to examine whether co-treatment of a corticosteroid and/or aprepitant influenced the frequencies of the SEs.

In summary, our present results demonstrated that combination of a corticosteroid with a 5-HT_3_ receptor antagonist significantly improved the CINV control in patients receiving ABVD therapy, whereas the differences between granisetron and palonosetron and between presence and absence of aprepitant did not influence that. These findings suggested that CINV in patients with Hodgkin lymphoma receiving ABVD therapy could be controlled more effectively by a granisetron-based antiemetic regimen in combination with a corticosteroid, although the careful management of febrile neutropenia is required.

## Conclusion

These findings suggested that a combination use of a corticosteroid with a 5-HT_3_ receptor antagonist was preferable for CINV control in patients with Hodgkin lymphoma receiving ABVD therapy.

## Abbreviations

5-HT_3_: 5-Hydroxytryptamine; 5-HT type 3
ABVD: Adriamycin, Bleomycin, Vinblastine, and Dacarbazine
ADEs: Adverse drug events
CC: Complete response
CINV: Chemotherapy-induced nausea and vomiting
CR: Complete response
CTCAE: Common Terminology Criteria for Adverse Events
CYP: Cytochrome P450
ECOG-PS: Eastern Cooperative Oncology Group performance status
HEC: Highly emetogenic chemotherapy
MEC: Moderately emetogenic chemotherapy
SEs: Side effects
